# A novel, rapid, seedless, *in situ* synthesis method of shape and size controllable gold nanoparticles using phosphates

**DOI:** 10.1038/s41598-019-43921-0

**Published:** 2019-05-15

**Authors:** Kangze Liu, Zhonglei He, James F. Curtin, Hugh J. Byrne, Furong Tian

**Affiliations:** 1 Environmental Sustainability and Health Institute, Technological University Dublin, Grangegorman, Dublin, 7 Ireland; 2 School of Food Science and Environmental Health, College of Sciences and Health, Technological University Dublin, Cathal Brugha Street, Dublin, 1 Ireland; 3 FOCAS Research Institute, Technological University Dublin, Kevin Street, Dublin, 8 Ireland

**Keywords:** Nanoparticles, Synthesis and processing, Structural properties, Molecular self-assembly

## Abstract

We hereby report a novel synthesis method of size and shape controllable gold nanoparticles that is rapid, *in situ* and seedless. Unlike most currently employed size and shape controllable synthesis methods, it takes place in a single step under room temperature within ~15 minutes. While mixtures of gold nanospheres around 70 nm and gold nanoplates with width ranging from 100 nm to 1000 nm can be synthesized in about 15 minutes by standard synthesis method using *N*-2-hydroxyethylpiperazine-*N*-2-ethanesulphonic acid (HEPES) to reduce Au(III), gold nanoflowers or mixtures of smaller gold nanospheres and nanoplates can be synthesized with the addition of disodium phosphate (Na_2_HPO_4_) or monosodium phosphate (NaH_2_PO_4_), respectively. Increasing the concentration of phosphate added significantly reduces the formation time of gold nanoparticles to seconds. By increasing the molar ratio of Na_2_HPO_4_: HEPES and NaH_2_PO_4_: HEPES, the size of gold nanoflowers and gold nanoparticle mixtures can be tuned from ~60 nm down to 1 nm and from ~70 nm to ~2.5 nm, respectively. The systematic structural changes are accompanied by similarly systematic colour changes associated with shifting of the surface plasmon resonance. The proposed mechanism of the synthesis process is also presented.

## Introduction

Gold nanoparticles (GNPs) have received increasing attention as nanotechnology has flourished^1–5^. Their peculiar physical and chemical properties, especially the localized surface plasmon resonance (LSPR)^6,7^, has enabled them to be widely applied in diverse fields such as surface enhanced Raman scattering (SERS)^8,9^, chemical sensing^10^, and biomedicine^11^. The characteristics of the LSPR are dependent on the nanoparticle size, shape, composition, and distance and electric environment between nanoparticles, all of which can strongly influence the resonant frequency, which determines the characteristic colour, observable by the naked eye^12^. The electronic properties and associated colour can be employed for a number of application, and therefore, continued refinement of synthesis methods which can control the size and shape of GNPs is desirable.

Current synthesis methods of GNPs include ‘top-down’^13^ and ‘bottom-up’^14^ procedures. Top-down procedures lack control of size, shape and functionalization of the GNPs synthesized, whereas, in contrast, bottom-up procedures can often accomplish these, since they involve either chemical or biological reduction and assemble the particles from molecules^13,14^. The bottom-up procedure can be divided into two kinds: the seed-growth method, which can better control the size and shape of the GNPs, yet requires at least two processes since the nucleation and successive growth processes are separate; and the *in situ* method, which entails only one process^15^. To date, in addition to nanospheres^16^, GNPs with different shapes such as nanorods^17,18^, nanoplates^19–22^, planar nanoparticles^23,24^, and branched nanostructures like nanoflowers^25,26^ and nanostars^27,28^ have been synthesized. Among these shapes, the nanoplates have attracted particular attention due to the high local electric field gradients under illumination caused by their sharp edges, which can be applied in SERS^29^, and the nanoflowers, with rough surface and dense tips, are able to enhance SERS to a greater degree, and thus have been considered powerful SERS probes^26,30–32^.

On the other hand, the reaction between phosphates and GNPs has also attracted attention, especially in the use of GNP-based phosphate detection^33,34^. Organic pollutants in water, due to either natural or artificial processes, need to be monitored constantly to safeguard the supply of clean drinking water to the public, and to control the impact on the environment and the ecosystem^35^. The preferred assay among all detection methods is the colorimetric assay due to its speed and ease of observation of the results. For example, Yongdoo Choi *et al*. designed an assay by which phosphates will react with phosphorylated peptide modified GNPs, resulting in the aggregation of GNPs and thus a colour change from red to blue^36^. Other kinds of GNP-based phosphate detection methods have also been reported, such as the phosphate anion detection assay designed by Massue *et al*., which gives the results by the releasing of naked-eye-visible red Eu(III) after phosphate anions combines with modified GNPs^37^. However, although there have been many studies on phosphate interactions with modified GNPs, little attention has been paid to interactions between phosphates and unmodified GNPs, or the effect of phosphates on the synthesis process of GNPs itself.

In this study, comparing to the standard synthesis method of N-2-hydroxyethylpiperazine-N-2-ethanesulphonic acid (HEPES) reduced GNPs, which uses only HEPES and chloroauric acid^38,39^, a novel bottom-up *in situ* synthesis method using HEPES, chloroauric acid and different concentrations of monosodium phosphate or disodium phosphate is reported, which is seedless and can be accomplished under room temperature within 15 minutes. The GNPs synthesized can be size tuneable mixtures of gold nanospheres and nanoplates, or gold nanoflowers. By adjusting the molar ratio of phosphate to HEPES, the size of nanoflowers can be modulated from ~60 nm to less than 1 nm.

## Results and Discussions

### Estimated GNP formation time

The HEPES reduced GNP synthesis method has been applied and studied for a decade, and it has been reported that the difference of the molar ratio of chloroauric acid and HEPES strongly affects the GNP synthesized^38^. In this study, a molar ratio of 1:10, chloroauric acid to HEPES, has been applied to all syntheses, with or without phosphate added.

**Figure 1:**
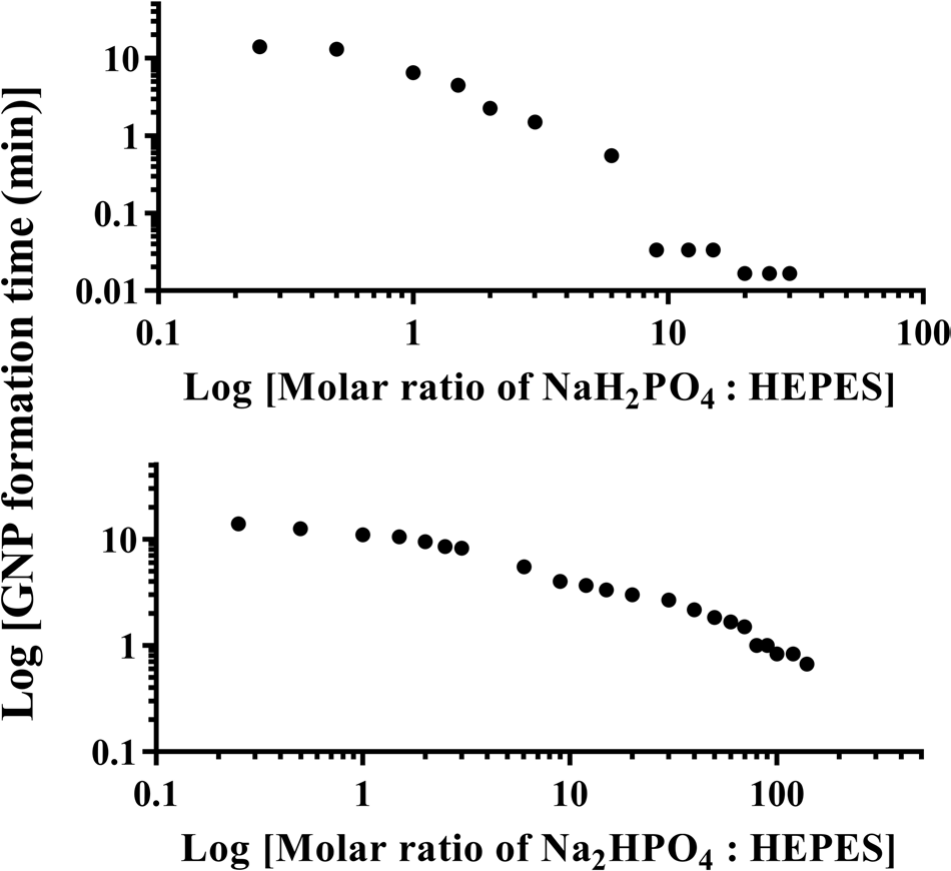
Estimated GNP formation times with different molar ratio of NaH_2_PO_4_ (top) and Na_2_HPO_4_ (bottom) to HEPES added.

During the synthetic progress of all colloidal gold samples, the colour of the system changed from pale yellow, which is the colour of chloroauric acid, into colourless, then became pink/purple/blue, representing the formation of GNPs. The colour changes of the colloidal systems were sufficient to be observed by naked eyes, thus the time from the start of the synthesis to the time at which the colour changed into pink/purple/blue, observed by the naked eye, was recorded as the estimated formation time of the GNP. The estimated formation times, shown in Fig. 1, were recorded to demonstrate the dependence of the rate of GNP formation on the concentration of NaH_2_PO_4_ (Fig. 1 top) or Na_2_HPO_4_ (Fig. 1 bottom) added.

While the estimated formation time of Standard GNP was 15 minutes (±30 seconds), with increased concentration of phosphate, shorter formation time was needed. Note, the estimated GNP formation time became less than 2 seconds when the molar ratio of NaH_2_PO_4_ to HEPES was higher than 9, as the colour changed instantaneously after the reaction started; and the estimated formation time stabilized at ~40 seconds when the molar ratio of Na_2_HPO_4_ to HEPES was higher than 100. As discussed further in a later section, the dynamics of GNP formation, and the differences for the different reaction systems, are complex and of considerable interest, and will be the subject of a more comprehensive future study.

### Optical properties

**Figure 2:**
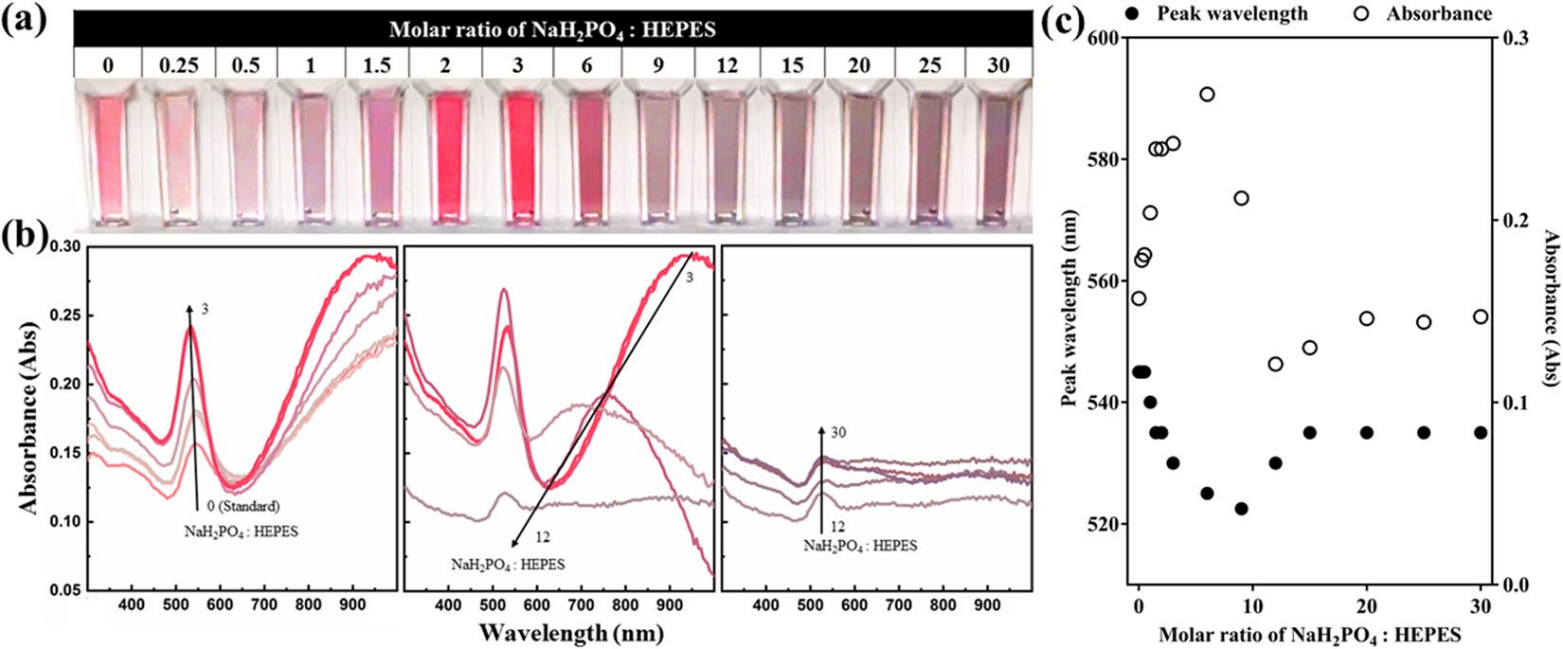
(**a**) Photograph and (**b**) UV/VIS/NIR spectrum of GNPs synthesized with different molar ratios of NaH_2_PO_4_: HEPES. Curves in (**b**) are coloured according to the colour in (**a**) with corresponding NaH_2_PO_4_: HEPES molar ratio. Arrows in (**b**) present the trended change of absorbance peak as the molar ratio changes. (**c**) The wavelength peak and corresponding absorbance change of first peak (wavelength smaller than 550 nm) as a function of NaH_2_PO_4_: HEPES molar ratio.

**Figure 3:**
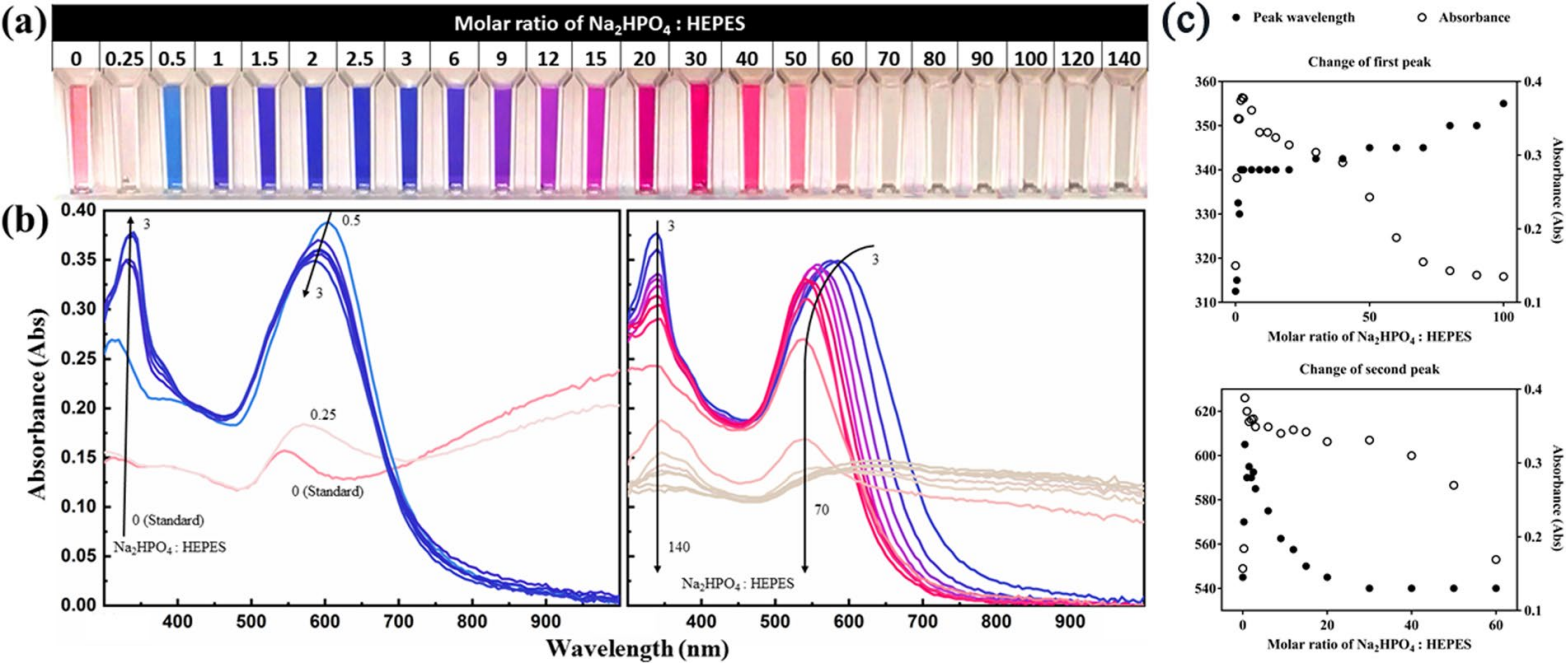
(**a**) Photograph and (**b**) UV/VIS/NIR spectrum of GNPs synthesized with different molar ratios of Na_2_HPO_4_: HEPES. Curves in (**b**) are coloured according to the colour in (**a**) with corresponding Na_2_HPO_4_: HEPES molar ratio. Arrows in (**b**) present the trended change of absorbance peak as the molar ratio changes. (**c**) The peak wavelength and corresponding absorbance change of first peak (top) and second peak (bottom) as a function of Na_2_HPO_4_: HEPES molar ratio.

The optical characteristics and properties of the GNP suspensions, obtained at 1 hour after synthesis started, are depicted using a combination of photographs and UV/VIS/NIR spectra, as shown in Figs 2 and 3.

As shown in Figs 2 and 3, the colour and spectrum of the GNPs varied according to phosphate concentration, which has been converted to the molar ratio of phosphate to HEPES for better understanding.

While the colour of Standard GNP appeared pink, the colour of the ones with NaH_2_PO_4_ added became purple and pinkish red when the molar ratio of NaH_2_PO_4_: HEPES was in the range 1.5–6; and when the molar ratio was higher than 9, the colour turned to purple within seconds, then turned dark grey rapidly because of aggregation (Fig. 2(a)). The higher the molar ratio, the deeper the grey colour of colloidal GNPs became.

The corresponding UV/VIS/NIR spectra of the GNPs synthesized with NaH_2_PO_4_ also show systematic trends (Fig. 2(b,c)). Samples with molar ratio smaller than 12 exhibit 2 LSPR peaks, with one lower than 550 nm (first peak) and the other one higher than 700 nm (second peak). As the GNPs aggregate almost instantly when the molar ratio is higher than 9, the changing trend of spectra appeared different for GNPs with molar ratio higher and lower than 9.

When the molar ratio is smaller than 9, the wavelength of both the first and second peak decreases as the molar ratio increases, and the absorbance of GNPs appears strongest when the molar ratio is 6. On the other hand, when the molar ratio is larger than 9, the wavelength and absorbance of first peak increases along with the molar ratio, then tends to be stable with only slight increase, matching the denser colour, which is caused by the stronger aggregation and larger aggregates. The second peak disappears when the molar ratio is higher than 12, also due to aggregation.

In comparison, the GNPs synthesized with Na_2_HPO_4_ added appear bright blue when the molar ratio of Na_2_HPO_4_: HEPES is 0.5–6, and slowly changes to purple and pinkish red when the molar ratio is increased to 30, and then changes into light pink when the molar ratio reaches 60. When the molar ratio is 70 and higher, the colour of GNPs turns pink in seconds, then changes into blue and eventually to grey as the aggregation proceeds. The higher the molar ratio, the faster the aggregation proceeds, and the denser the final colour appears, as the aggregates are larger (Fig. 3(a)).

The UV/VIS/NIR spectra of this set of samples appears rather well behaved (Fig. 3(b)). A red-shift of the first peak (the peak smaller than 400 nm) is apparent when the molar ratio increases from 0 to 100, while the corresponding absorbance reaches its highest value when the molar ratio is 3, whereupon a blue-shift of the second peak (the one higher than 500 nm) occurs as the molar ratio increases from 0.5 to 60, along with the decrease of corresponding absorbance, matching the colour change from blue to red/pink. Similar to GNPs synthesized with NaH_2_PO_4_ added, the absorbance peaks disappear at high molar ratio because of aggregation.

### Crystallinity, size and morphological properties

The size and zeta potential of GNPs synthesized with different molar ratios of phosphate to HEPES were measured using a Zetasizer at ~40 minutes after synthesis started, as presented in Table 1. The average size, calculated from the Size-Number distribution, show trends that match the spectral results. This is consistent with the reports by Haiss *et al*. that the size and concentration of GNPs within the size range of 5–100 nm can be directly determined from UV-VIS spectra according to corrected Mie theory^40^. The size reaches 350–700 nm when the molar ratio of NaH_2_PO_4_: HEPES is higher than 9, and Na_2_HPO_4_: HEPES is larger than 60, which is because of the rapid aggregation. While Standard GNPs have an average size of ~62 nm, small particles ranging between 1.5–6.5 nm are synthesized when NaH_2_PO_4_: HEPES reaches 1.5–6; and ultra-small particles down to 0.5–3.5 nm are synthesized when the molar ratio of Na_2_HPO_4_: HEPES is 1–12. The trended change of absolute values of zeta potentials is caused by the change of pH due to the increase of phosphate concentration. The measurements of zeta potential values were made to monitor the stability of GNP samples, and the fact that no abrupt changes in zeta potential values was observed is an indication of the stability of the nanoparticles in suspension.

**Table 1:** Average size, zeta potential and pH values of GNPs synthesized at different molar ratios of phosphate to HEPES. Results presented in table are mean ± standard error of the mean.

Molar ratio of phosphate to HEPES	With monosodium phosphate added			With disodium phosphate added		
	Size [nm]	Zeta potential [−mV]	pH	Size [nm]	Zeta potential [−mV]	pH
0 (Standard)	61.69 ± 22.35	23.50 ± 0.21	3.8	61.69 ± 22.35	23.50 ± 0.21	3.8
0.25	313.45 ± 106.80	25.27 ± 0.08	3.4	59.27 ± 14.62	25.77 ± 0.16	4.3
0.5	46.90 ± 10.04	30.73 ± 0.10	3.4	52.26 ± 14.94	40.80 ± 0.24	5.8
1	82.33 ± 35.86	26.43 ± 0.13	3.4	2.40 ± 0.67	41.17 ± 0.24	6.5
1.5	2.74 ± 1.24	28.70 ± 0.14	3.4	2.67 ± 0.55	39.07 ± 0.45	6.8
2	4.56 ± 1.70	28.93 ± 0.23	3.4	2.05 ± 0.43	41.83 ± 0.17	6.9
2.5				1.61 ± 0.33	36.10 ± 0.58	7.1
3	4.60 ± 1.32	29.67 ± 0.13	3.6	1.42 ± 0.32	34.97 ± 0.35	7.1
6	2.64 ± 0.70	30.90 ± 0.25	3.6	0.98 ± 0.21	32.47 ± 0.17	7.4
9	425.50 ± 165.19	32.83 ± 0.33	3.7	0.88 ± 0.17	36.30 ± 1.04	7.6
12	644.25 ± 205.58	33.30 ± 0.33	3.8	0.71 ± 0.15	31.77 ± 0.38	7.8
15	664.33 ± 191.90	35.67 ± 0.71	3.9	18.01 ± 4.69	30.67 ± 0.20	7.8
20	634.54 ± 224.30	36.87 ± 0.45	3.9	15.89 ± 4.42	26.50 ± 0.22	8.0
25	633.01 ± 177.46	37.77 ± 0.74	4.0			
30	611.70 ± 262.53	38.93 ± 0.72	4.0	15.91 ± 4.66	28.67 ± 0.09	8.2
40				18.39 ± 3.87	27.03 ± 0.21	8.3
50				288.23 ± 98.29	27.17 ± 0.50	8.4
60				373.05 ± 107.22	24.53 ± 0.11	8.5
70				363.21 ± 109.21	23.90 ± 0.33	8.5
80				383.34 ± 143.74	23.40 ± 0.38	8.6
90				483.70 ± 171.96	22.73 ± 0.31	8.7
100				507.81 ± 168.18	21.57 ± 0.32	8.7
120				391.05 ± 153.47	22.13 ± 0.27	8.8
140				477.02 ± 195.44	22.93 ± 0.29	8.8

The average size was calculated from Size-Number distribution, and average zeta potential was calculated from triplicate results. All size and zeta potential results were measured at ~40 minutes after synthesis started.

To further understand the properties of GNPs synthesized with or without phosphate, representative samples were chosen to be analysed using XRD, SEM and TEM.

The XRD pattern for GNPs synthesised by all 3 methods are presented in Fig. S1. Standard GNP and GNP synthesized with disodium phosphate added (molar ratio Na_2_HPO_4_: HEPES = 3) exhibited the same characteristic Au crystalline pattern of peaks at 2θ = ~38.5°, ~45.2°, ~65.2°, and ~77.94°, which correspond to Au (111), (200), (220) and (311) Bragg reflections, indicating the formation of GNPs with a face-centred-cubic (fcc) lattice structure (lattice constant = 0.407 nm)^41–43^. The XRD pattern for GNP synthesized with monosodium phosphate added (molar ratio NaH_2_PO_4_: HEPES = 3) is slightly different to that of the samples of the other two methods. Although the characteristic Au (111), (200), (220) and (311) peaks are discernible, stronger peaks appear at 2θ = ~32.04°, ~56.74°, and ~66.44°. It has been reported that, while most GNPs crystallize in a fcc lattice structure, tetragonal phase and orthorhombic phases can also be produced^44,45^. Su *et al*. have discussed the importance of different crystal planes on the surface of polyhedral GNPs^46^, and GNP synthesized by biomass of *Aspergillus terreus* have been shown to exhibit XRD pattern non characteristic of fcc Au^47^. The peaks of the GNP sample synthesized with monosodium phosphate added can be assigned to Au (110), (211), and (220) Bragg reflections of a strained fcc (lattice constant = 0.397 nm) or tetragonal crystal structure of the surface, with a fcc core. Further study will be carried out to further investigate the differences in gold crystalline structure due to the different synthesis methods.

**Figure 4:**
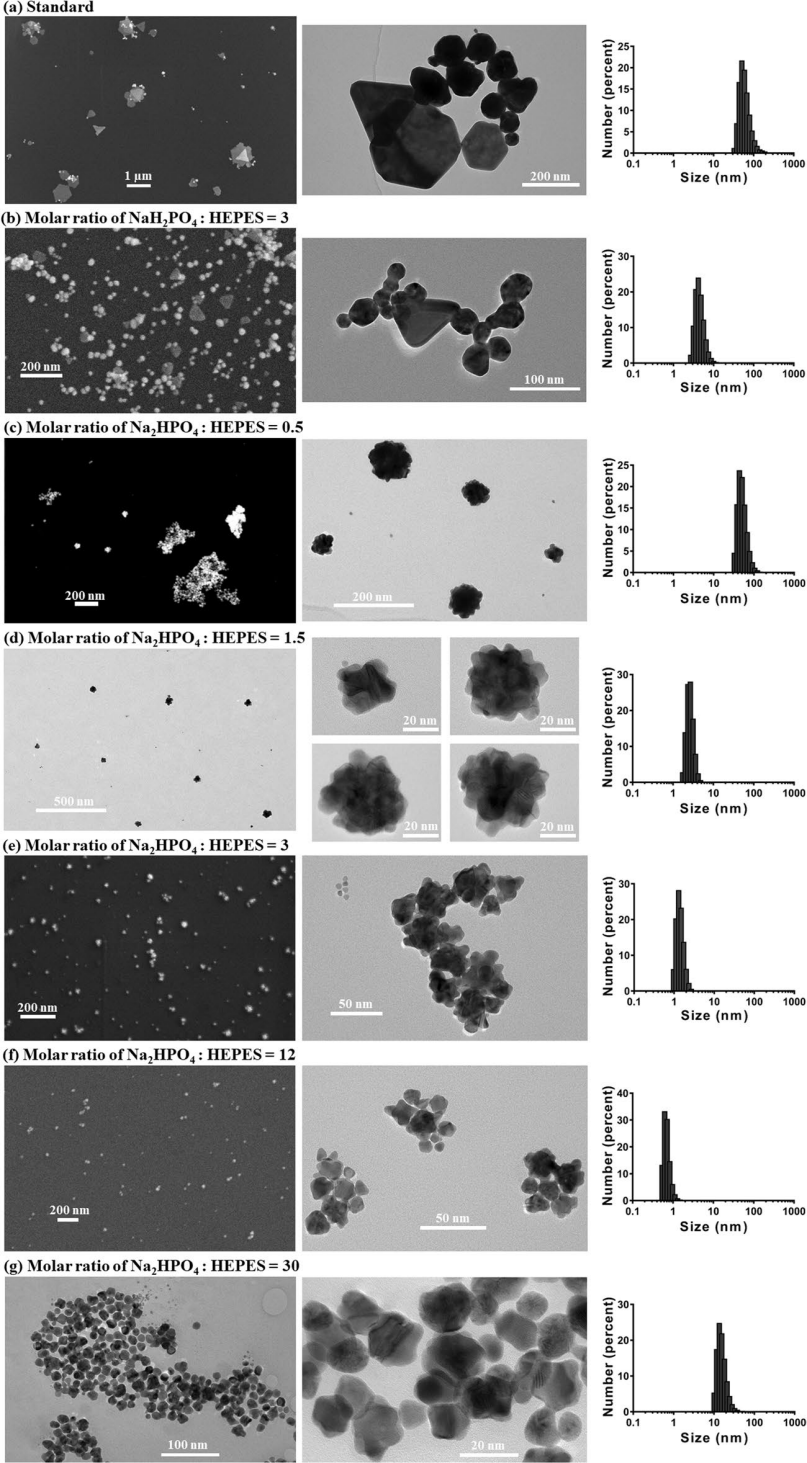
SEM/TEM images of representative samples, along with corresponding size-number distribution histogram. Samples presented are: (**a**) Standard; (**b**) GNP synthesized with NaH_2_PO_4_ added, the molar ratio of NaH_2_PO_4_: HEPES = 3; (**c**–**g**) GNPs synthesized with Na_2_HPO_4_ added, at molar ratios of Na_2_HPO_4_: HEPES = 0.5, 1.5, 3, 12, 30, respectively. SEM/TEM images were taken between 1–2 hours since synthesis started, and the size-number distribution histogram was measured at ~40 minutes since synthesis started.

The SEM and TEM images along with corresponding Size-Number distribution are shown in Fig. 4.

The GNPs synthesized using the standard method and with NaH_2_PO_4_ added are mixtures of nanospheres and nanoplates, which matches and explains the 2 peaks of the corresponding spectral results. With the presence of nanoplates, a longitudinal surface plasmon resonance at higher wavelength and a transverse surface plasmon resonance at lower wavelength are both created^22,48–51^. Thus, for Standard GNP and GNPs synthesized with NaH_2_PO_4_ added, the first peak is attributed to the spherical GNPs, while the second peak is attributed to the gold nanoplates. When the molar ratio of NaH_2_PO_4_: HEPES is smaller than 9, a blue-shift of the first peak appears because the diameter of the spherical GNPs decreased (Fig. 1(c), Table 1)^40,52^. The significant blue-shift of the second peak, which decreased from greater than 1000 nm to ~700 nm, is caused by the size decrease of the nanoplates. As shown in Fig. 4(a,b), the size of the nanoplates of Standard GNP ranges from ~100 nm to ~1000 nm, while the nanoplate size is smaller than 100 nm when the molar ratio of NaH_2_PO_4_: HEPES was 3. When the molar ratio of NaH_2_PO_4_: HEPES was higher than 9, significant aggregation occurred rapidly, whereby gold nanospheres and nanoplates accumulated into large aggregates, and thus, the second peak disappears.

On the other hand, the GNPs synthesized with Na_2_HPO_4_ added appear to be gold nanoflowers grown from spherical cores into flowers with petals attached (Fig. 4(c–g)). According to Table 1, the size of GNPs decreases down to less than 1 nm as the molar ratio of Na_2_HPO_4_: HEPES increases from 0.5 to 12. When the molar ratio is 15–40, the size remains between 15 nm to 20 nm. Then when the molar ratio is larger than 50, aggregation occurs rapidly, resulting in large aggregates. In addition to the size change, the number of petals on each nanoflower also decreases as the molar ratio of Na_2_HPO_4_: HEPES increases, which can be seen by comparing the central images of Fig. 4(c–g). While the gold nanoflowers are entirely covered with petals when the molar ratio is less than 3, the petals do not cover the whole surface of the cores when the molar ratio is higher than 3, leading to non-spherical shapes. As the molar ratio increases to greater than 15, only a few petals can be found on each core. The change of GNP shape and size explains the change of the second peak of the corresponding spectral results shown in Fig. 3: when the molar ratio increases from 0.5 to 12, the size decrease of the nanoflowers leads to the blue-shift of the second peak; and when the molar ratio is between 15–40, the LSPR peak wavelength undergoes no further change since the GNP size does not change much. Similarly, stronger aggregation occurs when the molar ratio is further increased, causing large aggregates to sink, leading to the disappearance of the second peak and the decrease of absorbance.

### Formation processes of GNP samples

To further understand the synthesis process and mechanism of the GNP samples synthesized with or without phosphates added, representative samples were selected to be monitored using spectroscopy and dynamic light scattering for 48 hours after synthesis started, and SEM/TEM images were also taken within this period.

### Synthesis processes of GNPs with/without NaH_2_PO_4_ added

**Figure 5:**
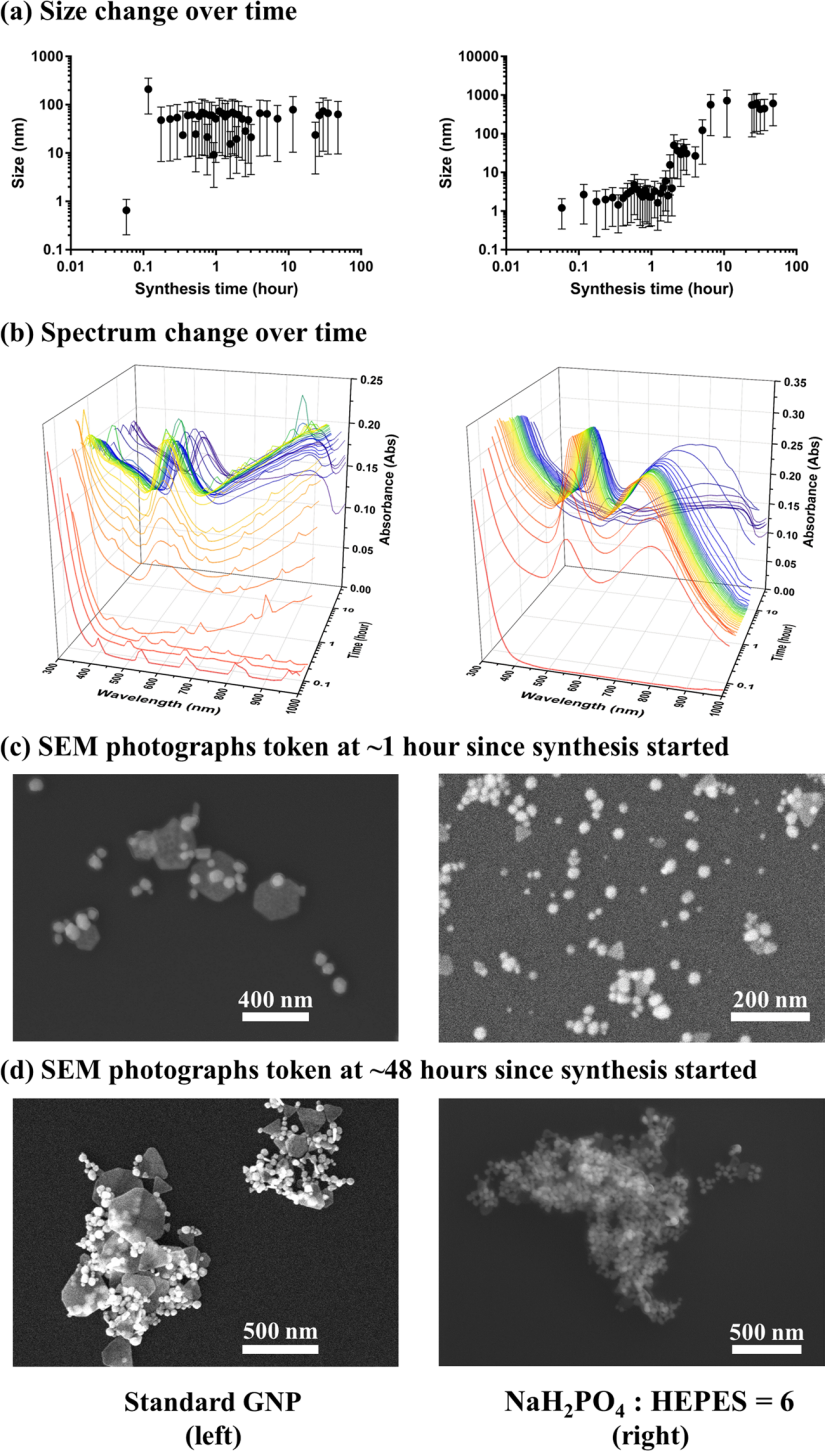
The (**a**) size change and (**b**) absorbance spectrum change over time, along with SEM photographs token at (**c**) ~1 hour and (**d**) ~48 hours since synthesis started, of Standard GNP (left) and GNP synthesized with the molar ratio of NaH_2_PO_4_: HEPES = 6 (right). The data points in A are average sizes calculated from Size-Number distribution measured using Zetasizer, and error bars are corresponding standard errors of the mean.

The Standard GNP samples are formed as a mixture of nanospheres and nanoplates. From Fig. 5(a,b), it can be found that the amount of same sized GNPs increases for ~40 minutes as the synthesis proceeds, since the GNP size stayed relatively stable and the spectral absorbance increased. After that, the GNPs kept stable until ~100 minutes after synthesis started, whereupon the GNPs started to aggregate, and the spectral absorbance decreased. The nanospheres and nanoplates appeared in small clusters before aggregation (Fig. 5(c) left), and assembled into large aggregates after aggregation (Fig. 5(d) left). For the first ~100 minutes of synthesis, the wavelength positioning of the longitudinal surface plasmon resonance peak created by the nanoplates was larger than 1000 nm, resulting in the rise of absorbance at 700–1000 nm. After ~100 minutes, the nanospheres started to assemble with nanoplates as aggregation started, decreasing the number of nanoplates, which led to the decrease of absorbance at 700–1000 nm.

For GNPs synthesized with NaH_2_PO_4_ added, the sample synthesized with the molar ratio of NaH_2_PO_4_: HEPES = 6 was selected as representative, and is referred to as Na6 GNP in the following sections. Similar to Standard GNP, the Na6 GNP was initially synthesized as a mixture of nanospheres and nanoplates, although the average particle size is significantly lower, which may support the proposal that the XRD pattern of Fig. S1 is dominated by the polyhedral surface rather than the Au fcc core. In the first ~20 minutes of synthesis, the number of same sized GNPs increased, and remained relatively stable for the following ~100 minutes. After ~120 minutes of synthesis, the aggregation started as the particle size gradually increased, while the second peak started to red-shift. This could be caused by the aggregation of nanoplates. It has been reported that the increase of the width of nanoplates would lead to a red-shift of the corresponding in-plane dipole plasmon absorption band^22,53–55^, so the red-shift in Fig. 5(b) (right) could be explained by the increase of nanoplate width, because of the aggregation. As the aggregation proceeds to ~400 minutes, the nanospheres and nanoplates assemble (Fig. 5(c) right) into large aggregates, as shown in Fig. 5(d) right, causing large particle sizes (Fig. 5(a) right) and the decrease of overall spectral absorbance (Fig. 5(b) right).

Overall, the synthesis processes of Standard GNP and GNP synthesized with NaH_2_PO_4_ added were similar: a mixture of gold nanospheres and nanoplates was synthesized, then aggregation began at different time points depending on the concentration of NaH_2_PO_4_ added. As shown in Fig. 1(b) top, the higher concentration of NaH_2_PO_4_ added, the faster the aggregation would happen.

### Synthesis processes of gold nanoflowers with Na_2_HPO_4_ added

**Figure 6:**
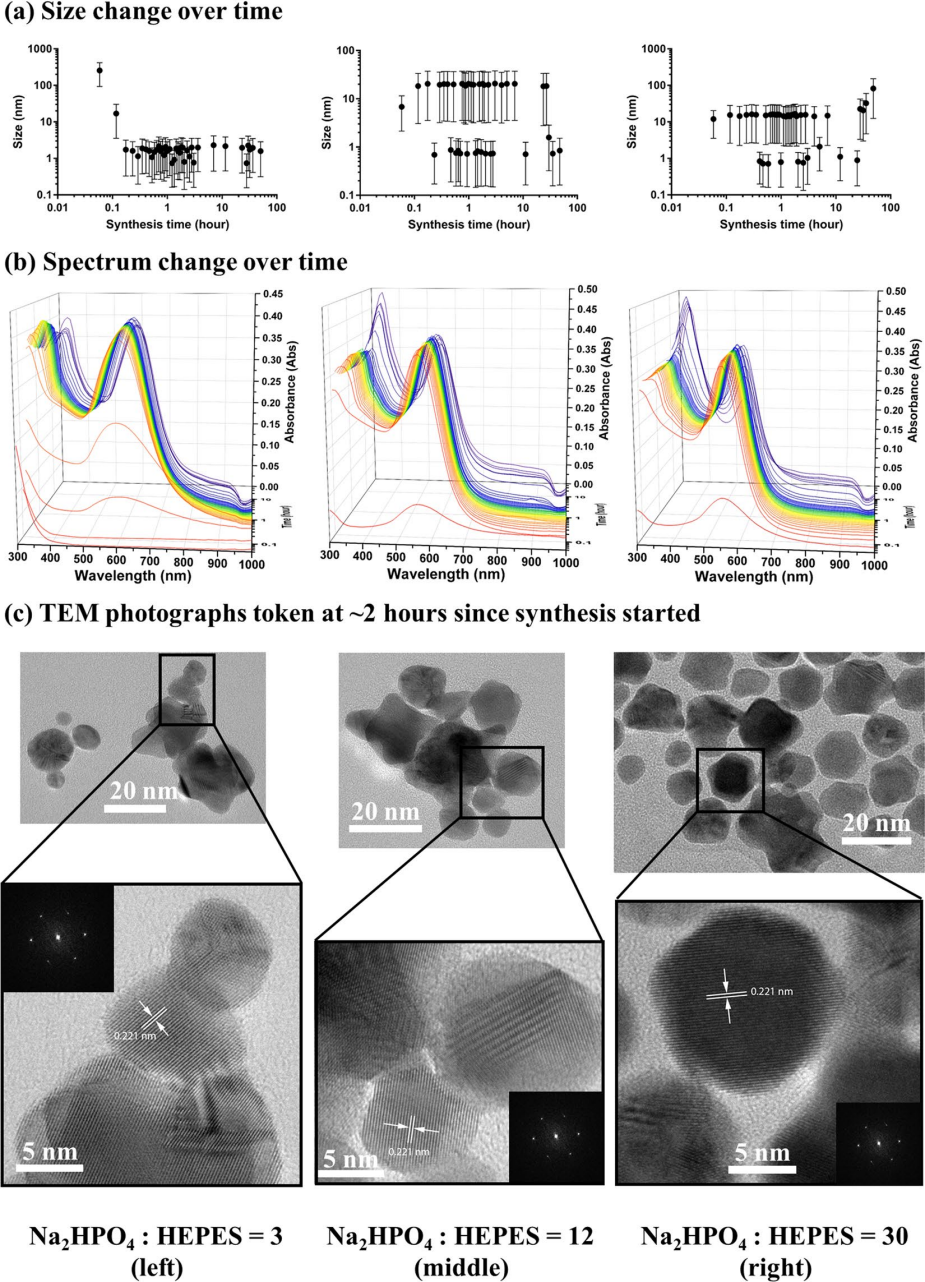
The (**a**) size change and (**b**) absorbance spectrum change over time, along with (**c**) low and high resolution TEM images and corresponding FFT images token at ~2 hours since synthesis started, of GNPs synthesized with the molar ratio of Na_2_HPO_4_: HEPES = 3 (left), 12 (middle) and 30 (right). The data points in A are average sizes calculated from Size-Number distribution measured using Zetasizer, and error bars are corresponding standard errors of the mean.

The GNPs synthesized with Na_2_HPO_4_ added were gold nanoflowers, as shown in Fig. 4. The samples synthesized with molar ratio of Na_2_HPO_4_: HEPES = 3, 12 and 30 were selected as representative, and will be referred to as Na_2_3, Na_2_12 and Na_2_30 GNP, respectively in the following sections. Similarly, the GNPs synthesized with Na_2_HPO_4_ added reached stable particle sizes within the first few minutes, whereupon the amount of particles increased until the synthesis process finished, leading to the increase of the spectral absorbance (Fig. 6(a,b)). The time of these synthesis processes varied with different concentrations of Na_2_HPO_4_ added. The higher concentration of Na_2_HPO_4_ added, the shorter the time needed for the synthesis process to come to completion (Fig. 1(b) bottom).

The spectra of these 3 samples appears rather interesting. While the second peaks of each sample, which are caused by the localized surface plasmon resonance of gold nanoflowers, stayed rather stable over time once synthesis was complete, the first peaks changed with the same trend. The first peak of Na_2_3 GNP appeared at 310 nm, after ~17 minutes of synthesis, then red-shifted to 335 nm while the absorbance increased at the same time until ~1 hour after synthesis started; then the red-shift continued rather slowly to 340 nm over the time, with absorbance decreasing slightly. Similarly, the first peaks of Na_2_12 and Na_2_30 GNP both appeared at 330 nm after ~10 minutes, then red-shifted to 340 nm with increased absorbance within 10 minutes. The peak of Na_2_12 GNP did not change until ~7 hours after synthesis, when the absorbance started to increase significantly. Similarly, the peak of Na_2_30 GNP stayed unchanged until ~2 hours after synthesis started, whence the absorbance increased abruptly; then the absorbance decreased slightly after ~24.5 hours of synthesis. On the other hand, the second peaks of Na_2_3, Na_2_12 and Na_2_30 GNP remained stable until ~11.5 hours, ~7 hours and ~5 hours after synthesis started, respectively, whence the absorbance started to decrease slightly because of the aggregation. Higher concentration of NaH_2_PO_4_ added caused faster aggregation of GNP samples as well.

From Fig. 6(c), it can be seen that the nanoflowers are grown from spherical cores into flowers, with smaller particles in the images demonstrating the uncomplete growth. Relating the UV/VIS/NIR spectral results of these gold nanoflowers as shown in Fig. 3, it should be noticed that, in contrast to common nanoflowers which have 2 LSPR peaks, located at 520–600 nm, and the other one, caused by aggregation, located at longer wavelength^56–59^, these nanoflowers synthesized with Na_2_HPO_4_ added only exhibit one LSPR band in the VIS/NIR region. This phenomenon, as previously reported by Hong Yuan *et al*. and Wei Wang *et al*., could be caused by the difference in synthesis mechanisms: instead of the aggregation between grown petals and cores, these nanoflowers were formed by a second growth on cores^60,61^. The cores grew from very small into relatively larger spherical particles, then a second growth started, resulting in the petals on the cores. The HRTEM and corresponding FFT images in Fig. 6(c) showed that the petals selectively grew in the <111> direction, and had same *d*-spacing with cores of 0.221 nm. The presence of larger flowers and smaller cores could also explain the 2 different average sizes of Na_2_12 and Na_2_30 GNP measured by Zetasizer in Fig. 6(a).

### Proposed formation scheme

**Figure 7:**
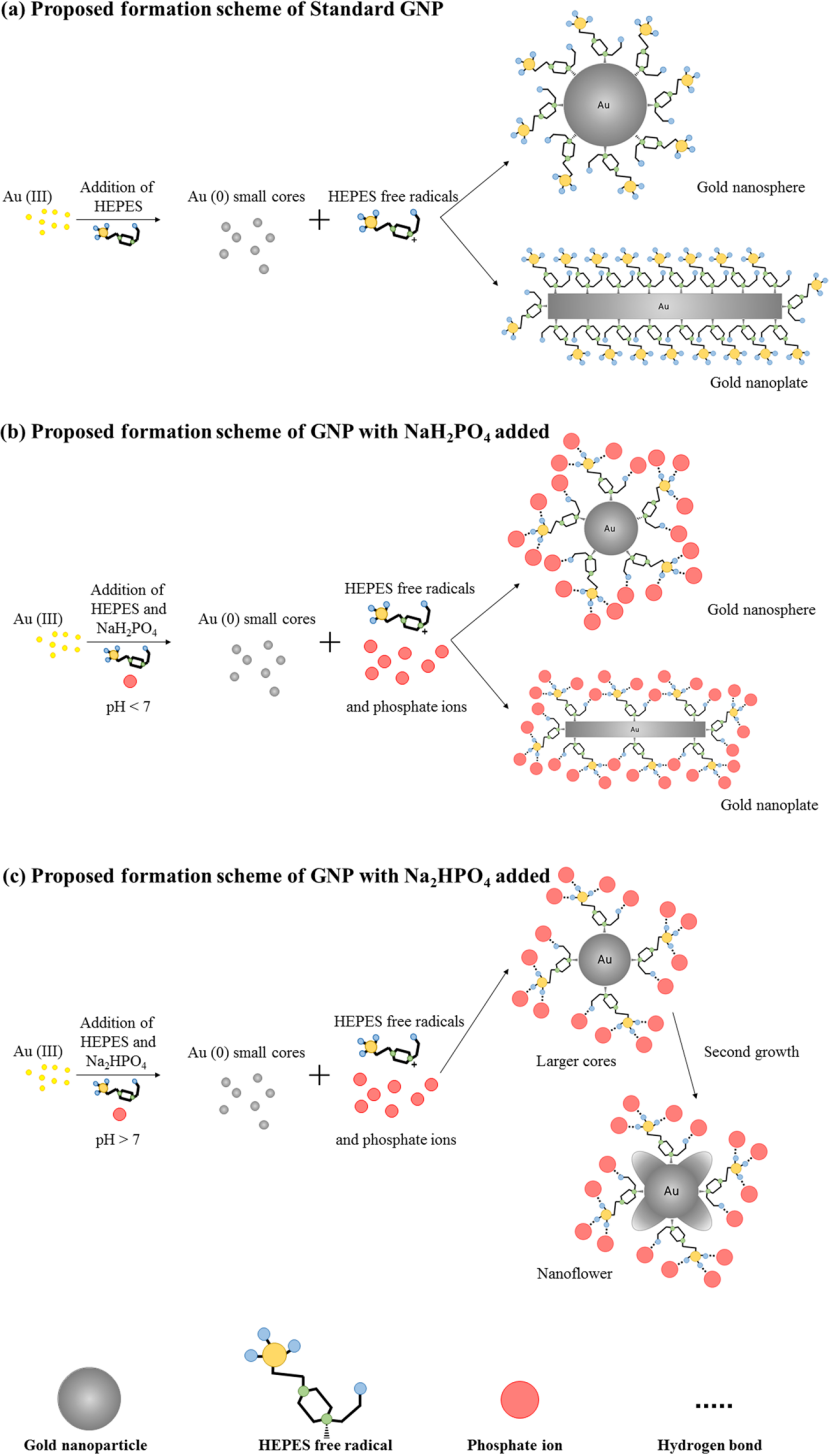
The formation schemes of (**a**) Standard GNP, (**b**) GNP synthesized with NaH_2_PO_4_ added, and (**c**) GNP synthesized with Na_2_HPO_4_ added.

As the different major facets of crystalline gold have similar chemistry and surface free energies, an exact mechanism of the shape control of GNPs has proven nontrivial to formulate^29^. We hereby propose possible formation schemes of GNPs with or without phosphates added during the synthesis processes, as shown in Fig. 7.

For a standard HEPES reduced synthesis of GNPs, the reaction process has been well documented^39,62^: HEPES reduces Au(III), creating Au(II) and cationic free radicals of HEPES; then the disproportionation of Au(II) creates Au(III) and Au(I); and Au(I) continues to react with HEPES, creating Au(0) and more cationic HEPES free radicals. The Au(0) assembles into very small gold nanoparticle cores first. Then, during the growth process, the HEPES free radicals attach to the surface of the particles, stabilizing them from aggregation and controlling the particle size, as they create a fixed volume of space in which the GNP can grow^63,64^. The proposed structures of gold nanospheres and nanoplates are shown in Fig. 7(a).

With the addition of NaH_2_PO_4_, phosphate ions are introduced to the reaction system. The phosphate ions form hydrogen bonds with the HEPES free radicals, as shown in Fig. 7(b) (nanosphere), creating effectively longer polymer chains attached to the particle surfaces. With larger attachments, the space for nanospheres to grow is decreased, leading to smaller size particles. Similarly, with the ability of forming more than one hydrogen bond, the phosphate ions can also link several HEPES free radicals, as shown in Fig. 7(b) (nanoplate). With large polymer chains attached, the width and thickness is restricted to a much smaller range, compared to the conditions for growth of Standard GNP. When the molar ratio of NaH_2_PO_4_: HEPES is lower than 9, the more NaH_2_PO_4_ added, the more phosphate ions are brought into the system, creating more hydrogen bonds and thus larger polymer clusters attached to GNPs. Thus, a higher molar ratio of NaH_2_PO_4_: HEPES leads to smaller particle sizes. When the molar ratio of NaH_2_PO_4_: HEPES is further increased, the overloading of ions in the system destroys the synthesis environment and leads to rapid formation and aggregation. A similar trend has been reported for the ascorbic acid reduced, CTAB stabilized, GNP synthesis system, whereby the addition of small concentrations of iodide ions changes the morphology of GNPs synthesized, from nanorods (mixed with nanospheres) into nanoplates (mixed with nanospheres), and the higher the concentration of iodide ions added, the smaller the size of GNPs synthesized^21^.

It has been reported that the pH value plays a very important role in the synthesis of GNPs, as it can change the shape and size of GNPs synthesized^39,65,66^. It has also been reported that, using the same synthesis method, nanorods or mixtures of gold nanospheres and nanoplates were synthesized when the pH was smaller than 7, and gold nanoflowers were synthesized when the pH was equal to and larger than 7^67,68^.

In our study, with the addition of Na_2_HPO_4_, the pH value of the system increased to larger than 7, creating an alkaline environment, which inhibited the formation of cationic HEPES free radicals. Thus, insufficient stabilizer ions were present in the system for the formation of nanoplates. Under these conditions, the phosphate ions create hydrogen bonds with HEPES free radicals, and large polymer clusters attach to the particle surface, restricting the growth of gold cores, as shown in Fig. 7(c). Under an alkaline environment, a second growth of petals happens on the cores, resulting in the nanoflowers. With the increase of phosphate ion amount, the size of attached polymer clusters increases, leaving less space for petals to grow, which explains the decrease of petal amount as the molar ratio of Na_2_HPO_4_: HEPES increases.

The first peak of these nanoflowers could be caused by the coordinating action between HEPES free radicals and gold under alkaline conditions. As previously reported, the absorption peak at 346 nm has been noticed for GNPs synthesized using HEPES and 2-morpholinoethanesul-fonic acid (MES), and was proposed to be caused by the formation of nitro-compounds^39^. The red-shift of this peak could be caused by the amount decrease of HEPES free radicals coordinated with gold, and the change in absorption was caused by the amount change of complexes. Additional analysis and further research on the mechanisms of GNP formation according to this method will be carried out to support the proposed scheme.

## Conclusion

In summary, we have developed a novel bottom-up *in situ* synthesis method of shape and size controllable GNPs. The method is rapid, taking less than 15 minutes, and doesn’t need any seed. The GNPs are HEPES reduced with phosphate concerted. With NaH_2_PO_4_ added, mixtures of gold nanospheres and nanoplates sizing from ~70 nm to ~2.5 nm could be synthesized by increasing the molar ratio of NaH_2_PO_4_: HEPES from 0 to 6. And by adding Na_2_HPO_4_ into the synthesis system, gold nanoflowers sizing from ~60 nm down to ultra-small as less than 1 nm could be synthesized, with the amount of petals decreases as the molar ratio of Na_2_HPO_4_: HEPES increases. The GNP formation time decreases as the concentration of phosphate added increases. The LSPR peaks of the gold nanoflowers could be tuned from 605 nm to 540 nm. With such small size and controllable morphology, this synthesis method holds great potential in the application of surface enhanced Raman scattering imaging for cells, biolabling, plasmonic waveguiding and so on.

## Methods

### Reagents

Hydrogen tetrachloroaurate(III) trihydrate (chloroauric acid, HAuCl_4_·3H_2_O) was purchased from Fisher Chemical, Ireland. *N*-2-hydroxyethylpiperazine-*N*-2-ethanesulphonic acid (HEPES) buffer was purchased from Hampton Research, US. Sodium phosphate monobasic (NaH_2_PO_4_) and disodium hydrogen phosphate (Na_2_HPO_4_) were purchased from Sigma Aldrich, Ireland. Deionized water, deionized using Elix® Reference Water Purification System from Millipore, Ireland, was used for all experiments and solution preparations.

### Synthesis of colloidal GNPs

A 1 mM stock solution of chloroauric acid and 10 mM stock solution of HEPES buffer was made by dissolving hydrogen tetrachloroaurate in water and diluting purchased HEPES buffer with water accordingly. Different concentrations of phosphate stock solutions were similarly prepared by dissolving NaH_2_PO_4_ and Na_2_HPO_4_ in water.

In a standard synthesis process of GNPs, 200 µL of 1 mM chloroauric acid were mixed with 200 µL of 10 mM HEPES buffer and 600 µL of water under room temperature. The colour changed from pale yellow to colourless, then gradually changed to pink within ~15 minutes, indicating the formation of GNPs. The GNPs synthesized by this method are referred to as Standard GNP in the following sections.

On the basis of standard process, in the synthesis processes with phosphate added, similar procedures were followed, except that 200 µL of 1 mM chloroauric acid were mixed with 200 µL of 10 mM HEPES buffer and 600 µL of phosphate stock solutions with systematically varied concentrations.

### Characterization of GNPs

X-Ray Diffraction (XRD) analysis was performed using a Siemens diffractometer with a monochromatic Cu-Kα_1_ source operated at 40 keV and 30 mA. Samples were deposited from suspension onto the sample holder and allowed to dry, until a uniform coating was achieved.

Scanning electron microscope (SEM) images of GNPs were recorded by a Hitachi SU6600 FESEM instrument, and transmission electron microscope (TEM) images of GNPs were recorded by a JEOL JEM-2100 Electron Microscope instrument. Samples prepared for SEM imaging were dropped onto silicon wafers and spun for 2.5 minutes to dry in the air for ~1.5 hours after the synthesis reaction started. Samples prepared for TEM imaging were dropped onto copper grid with ultra-thin carbon film to dry in the air for ~1.5 hours after the synthesis reaction started. An accelerating voltage of 20 kV was used for all samples for SEM imaging, and 200 kV was used for all samples for TEM imaging.

A Perkin Elmer Lambda 900 UV/VIS/NIR Spectrometer was used to measure the absorption spectra of the GNPs in the 300–1000 nm region. The absorption spectra were used to monitor the formation of GNPs, and to characterize and discriminate different samples.

A Zetasizer Nano ZS Analyser from Malvern Instruments, Worcestershire, UK was used to measure the hydrodynamic particle sizes and zeta potentials of different GNP samples.

A Thermo Scientific™ Orion™ 3-Star Benchtop pH Meter was used to measure pH values of the GNP samples.

## Data Availability

All data generated or analysed during this study are included in this published article (and its Supplementary Information files).

## Supplementary information

Supplementary Information

## References

[1] Toshima, N. Nanoscale Materials. *M*. *Liz-Marzan L*., *Kamat PV*, (*Eds*), *Kluwer Academic Pub*., *London* **444**, 79–96 (2003).

[2] Nagarajan R (2008). Nanoparticles: building blocks for nanotechnology. Nanoparticles: synthesis, stabilization, passivation, and functionalization.

[3] Rogach, A., Talapin, D., Weller, H. & Caruso, F. Colloids and colloid assemblies. *Edited by Frank Caruso*, 447 *Wiley-VCH Verlag GmbH & Co*. *KGaA*, *Weinheim* **448** (2004).

[4] Schmid, G. (Wiley-VCH, Weinheim).

[5] Sreeprasad, T. S. & Pradeep, T. In *Springer Handbook of Nanomaterials* 303–388 (Springer, 2013).

[6] Willets KA, Van Duyne RP (2007). Localized surface plasmon resonance spectroscopy and sensing. Annu. Rev. Phys. Chem..

[7] Petryayeva E, Krull UJ (2011). Localized surface plasmon resonance: nanostructures, bioassays and biosensing—a review. Analytica chimica acta.

[8] Lal S (2008). Tailoring plasmonic substrates for surface enhanced spectroscopies. Chemical Society Reviews.

[9] Maiorano G (2011). Monodispersed and size-controlled multibranched gold nanoparticles with nanoscale tuning of surface morphology. Nanoscale.

[10] Ma W (2013). A SERS active gold nanostar dimer for mercury ion detection. Chemical Communications.

[11] Yang X, Yang M, Pang B, Vara M, Xia Y (2015). Gold nanomaterials at work in biomedicine. Chemical reviews.

[12] Liz-Marzán LM (2006). Tailoring surface plasmons through the morphology and assembly of metal nanoparticles. Langmuir.

[13] Nguyen DT, Kim D-J, Kim K-S (2011). Controlled synthesis and biomolecular probe application of gold nanoparticles. Micron.

[14] Parab H, Jung C, Woo M-A, Park HG (2011). An anisotropic snowflake-like structural assembly of polymer-capped gold nanoparticles. Journal of Nanoparticle Research.

[15] Zhao P, Li N, Astruc D (2013). State of the art in gold nanoparticle synthesis. Coordination Chemistry Reviews.

[16] Qian X (2008). *In vivo* tumor targeting and spectroscopic detection with surface-enhanced Raman nanoparticle tags. Nature biotechnology.

[17] Kuo T-R (2010). Multiple release kinetics of targeted drug from gold nanorod embedded polyelectrolyte conjugates induced by near-infrared laser irradiation. Journal of the American Chemical Society.

[18] Kariuki VM, Panetier JA, Schulte Jr, Sadik OA (2016). Directional Templating Mechanisms of Anisotropic Nanoparticles Using Poly (pyromellitic dianhydride-p-phenylenediamine). The Journal of Physical Chemistry C.

[19] Millstone JE (2005). Observation of a quadrupole plasmon mode for a colloidal solution of gold nanoprisms. Journal of the American Chemical Society.

[20] Busbee BD, Obare SO, Murphy CJ (2003). An improved synthesis of high‐aspect‐ratio gold nanorods. Advanced Materials.

[21] Ha TH, Koo H-J, Chung BH (2007). Shape-controlled syntheses of gold nanoprisms and nanorods influenced by specific adsorption of halide ions. The Journal of Physical Chemistry C.

[22] Ah CS (2005). Size-controlled synthesis of machinable single crystalline gold nanoplates. Chemistry of Materials.

[23] Sau TK, Murphy CJ (2004). Room temperature, high-yield synthesis of multiple shapes of gold nanoparticles in aqueous solution. Journal of the American Chemical Society.

[24] Kou X (2007). Glutathione-and cysteine-induced transverse overgrowth on gold nanorods. Journal of the American Chemical Society.

[25] Jiang Y, Wu X-J, Li Q, Li J, Xu D (2011). Facile synthesis of gold nanoflowers with high surface-enhanced Raman scattering activity. Nanotechnology.

[26] Li Q (2013). High Surface‐Enhanced Raman Scattering Performance of Individual Gold Nanoflowers and Their Application in Live Cell Imaging. Small.

[27] Barbosa S (2010). Tuning size and sensing properties in colloidal gold nanostars. Langmuir.

[28] Pei Y, Wang Z, Zong S, Cui Y (2013). Highly sensitive SERS-based immunoassay with simultaneous utilization of self-assembled substrates of gold nanostars and aggregates of gold nanostars. Journal of Materials Chemistry B.

[29] Grzelczak M, Pérez-Juste J, Mulvaney P, Liz-Marzán LM (2008). Shape control in gold nanoparticle synthesis. Chemical Society Reviews.

[30] Xie J, Zhang Q, Lee JY, Wang DI (2008). The synthesis of SERS-active gold nanoflower tags for *in vivo* applications. ACS nano.

[31] Fang J (2010). Gold mesostructures with tailored surface topography and their self-assembly arrays for surface-enhanced Raman spectroscopy. Nano letters.

[32] Wang Z, Zhang J, Ekman JM, Kenis PJ, Lu Y (2010). DNA-mediated control of metal nanoparticle shape: one-pot synthesis and cellular uptake of highly stable and functional gold nanoflowers. Nano letters.

[33] Johnson, M. Detection of Inorganic Phosphate in Environmental Water Samples using a Terbium and Gold Nanoparticle-based FRET Chemosensor. (2017).

[34] Kim S, Eom MS, Seo SH, Han MS (2013). Highly sensitive gold nanoparticle-based colorimetric probe for phytate detection with high selectivity over various phosphate derivatives. Tetrahedron Letters.

[35] Wang C, Yu C (2013). Detection of chemical pollutants in water using gold nanoparticles as sensors: a review. Reviews in Analytical Chemistry.

[36] Choi Y, Ho NH, Tung CH (2007). Sensing phosphatase activity by using gold nanoparticles. Angewandte Chemie.

[37] Massue J, Quinn SJ, Gunnlaugsson T (2008). Lanthanide luminescent displacement assays: the sensing of phosphate anions using Eu (III)− Cyclen-conjugated gold nanoparticles in aqueous solution. Journal of the American Chemical Society.

[38] Chen R (2010). Fabrication of gold nanoparticles with different morphologies in HEPES buffer. Rare Metals.

[39] Habib A, Tabata M, Wu YG (2005). Formation of gold nanoparticles by good’s buffers. Bulletin of the Chemical Society of Japan.

[40] Haiss W, Thanh NT, Aveyard J, Fernig DG (2007). Determination of size and concentration of gold nanoparticles from UV− Vis spectra. Analytical chemistry.

[41] Yang G-W, Gao G-Y, Wang C, Xu C-L, Li H-L (2008). Controllable deposition of Ag nanoparticles on carbon nanotubes as a catalyst for hydrazine oxidation. Carbon.

[42] Krishnamurthy S, Esterle A, Sharma NC, Sahi SV (2014). Yucca-derived synthesis of gold nanomaterial and their catalytic potential. Nanoscale research letters.

[43] Sneha K, Sathishkumar M, Kim S, Yun Y-S (2010). Counter ions and temperature incorporated tailoring of biogenic gold nanoparticles. Process Biochemistry.

[44] José-Yacamán M, Miki-Yoshida M, Tehuacanero S, Zorrilla C (1994). On the crystal structure of nanosized gold particles. Nanostructured materials.

[45] Jena, P., Khanna, S. & Rao, B. *Physics and chemistry of finite systems: from clusters to crystals*. Vol. 374 (Springer Science & Business Media, 2013).

[46] Su D, Dou S, Wang G (2015). Gold nanocrystals with variable index facets as highly effective cathode catalysts for lithium–oxygen batteries. *NPG Asia*. Materials.

[47] Gurunathan B, Bathrinarayanan PV, Muthukumarasamy VK, Thangavelu D (2014). Characterization of intracellular gold nanoparticles synthesized by biomass of Aspergillus terreus. Acta Metallurgica Sinica (English Letters).

[48] Halas NJ, Lal S, Chang W-S, Link S, Nordlander P (2011). Plasmons in strongly coupled metallic nanostructures. Chemical reviews.

[49] Maier SA, Atwater HA (2005). Plasmonics: Localization and guiding of electromagnetic energy in metal/dielectric structures. Journal of applied physics.

[50] Maier SA (2001). Plasmonics—a route to nanoscale optical devices. Advanced materials.

[51] Morsin M, Mat Salleh M, Ali Umar A, Sahdan MZ (2017). Gold nanoplates for a localized surface plasmon resonance-based boric acid sensor. Sensors.

[52] Kelly, K. L., Coronado, E., Zhao, L. L. & Schatz, G. C. (ACS Publications, 2003).

[53] Wang L (2004). Controllable morphology formation of gold nano-and micro-plates in amphiphilic block copolymer-based liquid crystalline phase. Chemistry letters.

[54] Fan X (2010). Size-controlled growth of colloidal gold nanoplates and their high-purity acquisition. Nanotechnology.

[55] Huang W-L, Chen C-H, Huang MH (2007). Investigation of the growth process of gold nanoplates formed by thermal aqueous solution approach and the synthesis of ultra-small gold nanoplates. The Journal of Physical Chemistry C.

[56] Yang Z, Lin ZH, Tang C-Y, Chang H-T (2007). Preparation and characterization of flower-like gold nanomaterials and iron oxide/gold composite nanomaterials. Nanotechnology.

[57] Jena BK, Raj CR (2007). Synthesis of flower-like gold nanoparticles and their electrocatalytic activity towards the oxidation of methanol and the reduction of oxygen. Langmuir.

[58] Jena BK, Raj CR (2007). Shape-controlled synthesis of gold nanoprism and nanoperiwinkles with pronounced electrocatalytic activity. The Journal of Physical Chemistry C.

[59] Maye MM (2003). Size-controlled assembly of gold nanoparticles induced by a tridentate thioether ligand. Journal of the American Chemical Society.

[60] Yuan H (2007). Shape and SPR evolution of thorny gold nanoparticles promoted by silver ions. Chemistry of materials.

[61] Wang W, Cui H (2008). Chitosan-luminol reduced gold nanoflowers: from one-pot synthesis to morphology-dependent SPR and chemiluminescence sensing. The Journal of Physical Chemistry C.

[62] Habib A, Tabata M (2004). Oxidative DNA damage induced by HEPES (2-[4-(2-hydroxyethyl)-1-piperazinyl] ethanesulfonic acid) buffer in the presence of Au (III). Journal of inorganic biochemistry.

[63] Napper, D. H. *Polymeric stabilization of colloidal dispersions*. Vol. 3 (Academic Pr, 1983).

[64] Walker HW, Grant SB (1996). Coagulation and stabilization of colloidal particles by adsorbed DNA block copolymers: The role of polymer conformation. Langmuir.

[65] Si S, Mandal TK (2007). pH-controlled reversible assembly of peptide-functionalized gold nanoparticles. Langmuir.

[66] Ji X (2007). Size control of gold nanocrystals in citrate reduction: the third role of citrate. Journal of the American Chemical Society.

[67] Gericke M, Pinches A (2006). Biological synthesis of metal nanoparticles. Hydrometallurgy.

[68] Okitsu K, Sharyo K, Nishimura R (2009). One-pot synthesis of gold nanorods by ultrasonic irradiation: the effect of pH on the shape of the gold nanorods and nanoparticles. Langmuir.

