# Supplementary material for: A novel, rapid, seedless, in situ synthesis method of shape and size controllable gold nanoparticles using phosphates
Source: Sci Rep. 2019 May 15;9:7421. doi: 10.1038/s41598-019-43921-0 (PMC6520384; doi:10.1038/s41598-019-43921-0)
Supplement: Supplementary file 1 — Supplementary Information [file 41598_2019_43921_MOESM1_ESM.docx]

***Supplementary Information:***

**A novel, rapid, seedless, *in situ* synthesis method of shape and size controllable gold nanoparticles using phosphates**

Kangze Liu*^1, 2, 3^, Zhonglei He^1, 2, 3^, James F. Curtin^1, 2^, Hugh J. Byrne^3^ and Furong Tian^1, 2^

^1^Environmental Sustainability and Health Institute, Technological University Dublin, Grangegorman, Dublin 7, Ireland; ^2^School of Food Science and Environmental Health, College of Sciences and Health, Technological University Dublin, Cathal Brugha Street, Dublin 1, Ireland; and ^3^FOCAS Research Institute, Technological University Dublin, Kevin Street, Dublin 8, Ireland

**Corresponding author**

*Correspondence to Kangze Liu (kangze.liu@dit.ie).


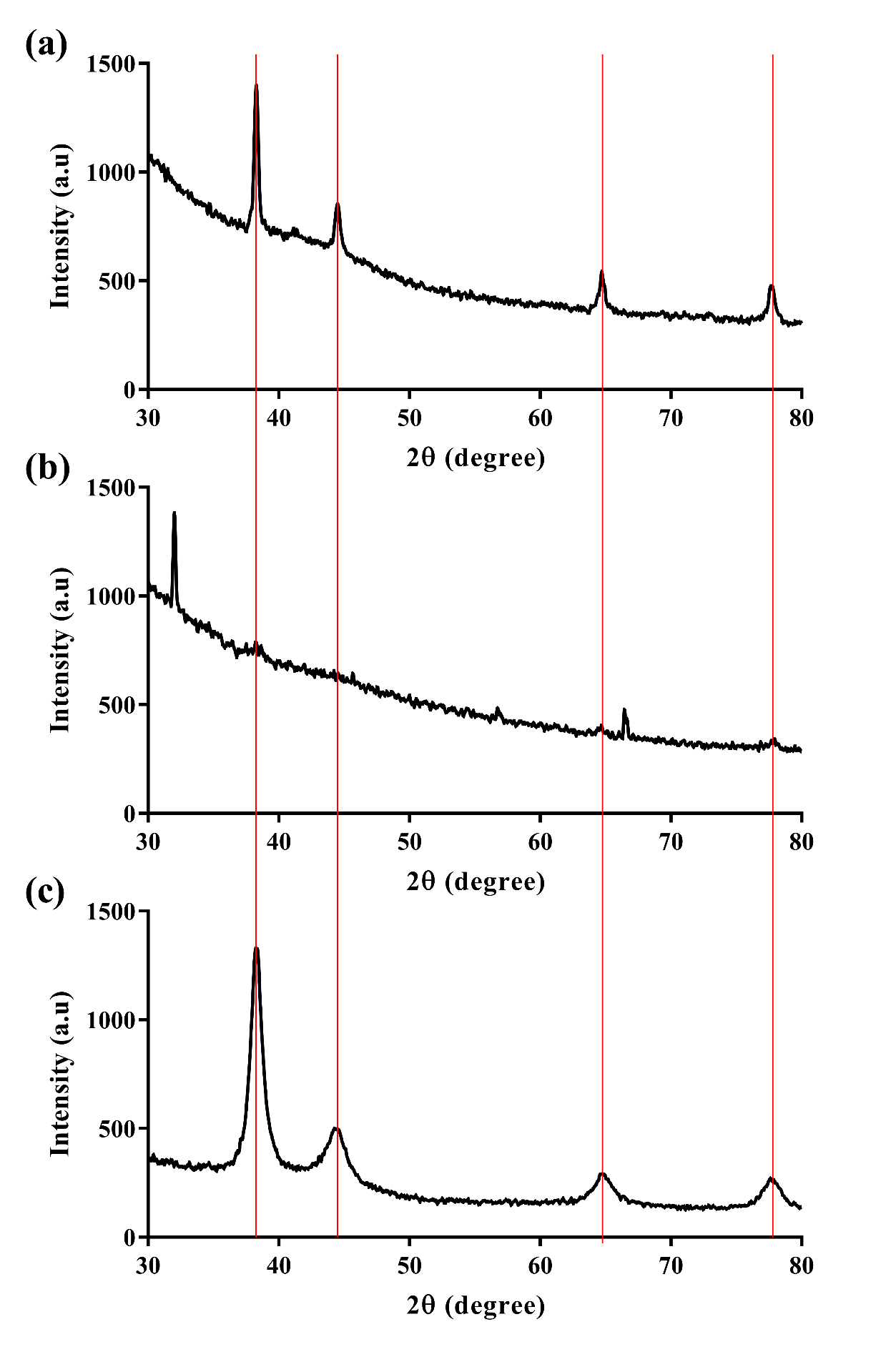


**Figure S1.** XRD analysis of GNP samples. Samples presented are: (a) Standard; (b) GNP synthesized with NaH_2_PO_4_ added, with a molar ratio of NaH_2_PO_4_ : HEPES = 3; (c) GNPs synthesized with Na_2_HPO_4_ added, at molar ratio of Na_2_HPO_4_ : HEPES = 3. The red lines indicate the fcc reflections corresponding to Au (111), (200), (220) and (311) Bragg planes.
